# The impact of barefoot racing in young Swedish trotters on career length

**DOI:** 10.1093/jas/skag127

**Published:** 2026-05-11

**Authors:** Paulina Berglund, Sreten Andonov, Erling Strandberg, Susanne Eriksson

**Affiliations:** Department of Animal Biosciences, Swedish University of Agricultural Sciences, 75007 Uppsala, Sweden; Department of Animal Biosciences, Swedish University of Agricultural Sciences, 75007 Uppsala, Sweden; Department of Animal Biosciences, Swedish University of Agricultural Sciences, 75007 Uppsala, Sweden; Department of Animal Biosciences, Swedish University of Agricultural Sciences, 75007 Uppsala, Sweden

**Keywords:** Swedish Standardbred trotter, Swedish-Norwegian Coldblooded trotter, survival analysis, performance, harness racing

## Abstract

In several European countries, racing without shoes, ie barefoot, is a common strategy in trotting races to improve the speed of the horse. Why some trotters can race barefoot without damage to the hoof from excessive wear and others cannot has been shown to partly be explained by differences in hoof composition. In particular, durable hind hooves are believed to be important to sustain racing without shoes. Also, variation in the proportion of the races the horse races with barefoot hind hooves has been shown to be affected by genetic differences in Swedish Standardbred trotters (SB) and Swedish-Norwegian Coldblooded trotters (CB). When racing barefoot, the protective properties of the shoe, which prevent the hoof from excessive wear and tear, are absent. If the hoof cannot withstand wear and tear, the damage of the hoof may pose a risk to animal welfare. The question of how barefoot racing should be regulated, especially for young horses, is under discussion in Sweden and elsewhere in Europe. Regulations regarding barefoot racing in young horses differ between countries, and there is a lack of published studies to base regulations on. Therefore, this study aimed to analyze the effect of racing with barefoot hind hooves in young SB and CB on career length. Datasets including shoeing information from 3-year-old SB, and 3- and 4-year-old CB were analyzed, including up to 12,161 horses. Cox proportional hazard models were fitted to analyze the effect of proportion of barefoot races at a young age on the hazard of ending the career at a given time point. Other effects such as sex, year of birth, started as a 2-year-old or not (SB only), early earnings, best racing time, and number of early starts were also included in the models. For SB, horses that raced more than 30% barefoot had a 14% higher hazard of ending their career at a given time point compared to the reference group that raced 5% or less barefoot. In CB, the hazard of ending the career at a given time point was 67% higher in the group with the highest proportion of barefoot races compared with the reference group. Although for SB, the reduction of the career length in days was minor. For CB, the effect was larger and verified the negative effect in SB but estimated based on fewer observations. Further studies of voluntary as well as involuntary reasons why Swedish trotters end their careers are needed to better understand the possible impact of racing barefoot as a young horse.

## Teaser

An increased knowledge of how barefoot racing in young trotters affects racing longevity could help to make informed decisions on new regulations. Having horses with long careers is not only of interest to stakeholders but also for animal welfare reasons and for maintaining the social license to operate in the harness racing industry.

## Introduction

Information about shoeing conditions has been recorded at Swedish race tracks since the end of 2004 and is an important aspect of betting because of its impact on racing speed (Solé et al. 2020). Whether or not young trotters should be allowed to race barefoot is a topic under discussion, and regulations differ between countries. Swedish Standardbred trotters (SB) can start racing from the 1st of July the year they turn two and Swedish-Norwegian Coldblooded trotters (CB) from 3 yr of age. However, barefoot racing is only allowed from 3 yr of age in both breeds (Svensk Travsport 2025a). In France, horses four years and older are permitted to race barefoot, and a maximum of 15 barefoot races over a period of 12 mo are allowed (The European Trotting Union 2024). A study on six French trotters (four barefoot and two shod) showed that the horses racing barefoot had an increased wear of the hoof wall and sole (Moiroud et al. 2014). The study also showed that horses racing barefoot had an increased sensitivity to pressure in the sole compared to shod horses, and that barefoot racing could cause inflammation in the distal phalanx. Animal welfare concerns regarding the increased wear and damage of the hooves and the possible impact on the durability of the horse if raced barefoot repeatedly have been mentioned as drivers for implementing restrictions on the intensity of barefoot racing in France (Le Trot 2024).

The Swedish Trotting Association has a self-inspection system that is part of the “Trotters’ welfare program” that should help ensure that the horse is in good condition to race before it enters a competition. The condition of the hoof is one of many control points included in the self-inspection checklist, where it is specified that if the horse should start in a race barefoot, the hoof needs to have enough hoof wall to sustain the wear and tear (Svensk Travsport 2024). Although no inspections are made upon entering the race with a barefoot horse, the trainer is held responsible and fined if a hoof is worn down to the point where the hoof is bleeding (Svensk Travsport 2025b). Besides this, horses at Swedish trotting races are subject to random inspections both before and after races. Since 2024, even more focus has been directed at inspections after races, where the horses are assessed on the impact on the wear of the hoof from racing barefoot to ensure good animal welfare (Svensk Travsport 2023a).

Previous studies on barefoot racing in Swedish trotters have been focused on the impact on performance, such as racing speed and risk of disqualification (Solé et al. 2020), understanding of the physical qualities of the hoof that is required for barefoot racing (Spörndly-Nees et al. 2023), as well as the genetic contribution to the ability to race barefoot (Berglund et al. 2025a; Schwochow et al. 2025). It is well known that being able to race barefoot is a trait of economic importance because it improves the speed, and Solé et al. (2020) showed that 0.7 s per kilometer can be gained by racing fully barefoot in Swedish Standardbred trotters. The same study also showed that racing with shod hind and barefoot front hooves improves speed but decreases the risk of breaking over to gallop and disqualification, compared with racing fully unshod. These results indicate that the hind hooves are more vulnerable to barefoot racing than the front hooves (Solé et al. 2020).

The ability to repeatedly race with barefoot hind hooves in Swedish trotters has been shown to be affected not only by environmental conditions but also by genetics, indicating potential to improve the ability to race barefoot repeatedly by selection (Berglund et al. 2025a). The heritability of the ability to repeatedly race barefoot, defined as the proportion of barefoot races, was estimated at 0.28 in SB and 0.21 in CB by Berglund et al. (2025a). For a second trait, barefoot status, which was a binary trait with repeated measurements, the heritability was estimated at 0.08 in SB and 0.07 in CB in the same study. The genetic correlation between the proportion trait and performance in SB and CB was favorable and moderate, while for barefoot status, it was weaker (low to moderate) (Berglund et al. 2025b). The traits proportion of barefoot races and barefoot status, defined by Berglund et al. (2025a) are derived from large datasets and could aid in selecting for improved ability to race barefoot in trotting races and indirectly improve, eg hoof quality and balance.

To pinpoint genomic regions important for hoof quality, Schwochow et al. (2025) analyzed tissue samples from the coronary band of the hind hooves for differences in gene expression (ie up- and downregulation of genes) in Swedish Standardbred trotters. The study showed that certain genes were differently expressed in horses reported to tolerate racing barefoot repeatedly without damage to the hoof than in horses that could not race barefoot without damage to the hooves. Downregulation of the gene *1-aminocyclopropane-1-carboxylate synthase-like protein* (ACCS) was found in horses reported to tolerate barefoot racing repeatedly without damage to the hoof. Schwochow et al. (2025) explained that the downregulation of ACC could decrease the ammonia content and thereby increase the ceramide content in the hoof. A decrease in ceramides has previously been linked to a softer horn in claws and laminitis in cows (Higuchi et al. 2005). Schwochow et al. (2025) reported four additional genes that were downregulated in horses reported to tolerate racing repeatedly barefoot without damage to the hoof: *Metallothionine* (MT2A), *trafficking protein particle subunit complex 6A* (TRAPPC6A), *iroquois homeobox 2* (IRX2), and *solute carrier family 35 member F3* (SLC3F3). The same authors argued that these genes were involved in pathways of importance for, eg keratin production, tissue and copper homeostasis in the hoof, and blood flow in the hoof, all of which play a role in protecting the hoof from wear.

While recent studies have shed light on the possibility of improving the ability to repeatedly race barefoot and hoof quality by including such traits in the genetic evaluation, it remains unknown whether and how repeated barefoot racing at a young age affects the expected career length in trotters. In the breeding goals of the SB and CB, it is specified that the horses should be durable and high-performing in trotting races, both as young and older individuals (Svensk Travsport 2025c). Therefore, if barefoot racing should be included in the genetic evaluation of Swedish trotters, the industry needs scientific evidence on how barefoot racing affects trotters’ chances of continued performance, also as older individuals.

The expected career length of trotters varies between studies. In the Finnish Standardbred trotter, the median career length was estimated at 3 yr in females and 4 yr in males (Aho 2025). In Australian Standardbred trotters and pacers, the median career length was estimated at 2.4 yr for horses starting their racing career as 3-year-olds Knight and Thomson (2011). Boorman et al. (2021) showed that American Standardbred trotters and pacers had a median career length of 2.8 yr. However, it should be noted that most horses included in the studies by Knight and Thomson (2011) and Boorman et al. (2021) were pacers, whereas in Sweden, there are no pacing races for Standardbreds (ie races performed in a lateral gait). The average career length was previously estimated to be 3.4 yr for Swedish SB (Árnason 2006) and 3.2 yr for CB (Velie et al. 2019). Factors previously shown to influence the expected career length in Swedish SB are: early performance, year of birth, sex, and inbreeding level (Árnason 2006), while for CB, sex, country of birth, and at what age the horse starts its racing career were shown to affect the career length (Velie et al. 2019).

Having horses that are competitive as older individuals is not only of interest to owners and trainers but also an important aspect of the acceptance of the sport by the general public. Therefore, this study aimed to analyze the effect of barefoot racing in young trotters on the career length in SB and CB, which could give guidance when deciding on new regulations to improve animal welfare in trotting races, avoiding a compromised durability of the horses. We hypothesized that the career length of Swedish trotters would be affected by proportion of barefoot races as a young horse, and by other factors, such as early performance, sex, and inbreeding level.

## Material and methods

### Data material

This study was based on routinely collected data from Swedish trotting races. In accordance with Swedish legislation, research based solely on the analysis of previously collected data does not require ethical approval from the regional animal ethics committee. Therefore, no ethical approval was required.

The data was provided by the Swedish Trotting Association and consisted of all racing records from Swedish races from 2005 to 2022 for SB and from 2005 to 2021 for CB. In 2005, recordings of shoeing information started in Swedish trotting races; hence, SB born from 2002 to 2018 and CB born from 2002 to 2017 were included in the study, as these could potentially have records of barefoot racing. For both breeds, horses born outside of Sweden were removed from the dataset. For CB, horses born in Sweden but registered in Norway were also excluded. Only horses born and registered in Sweden were considered, to prevent bias from including horses competing in Sweden for only a part of their career. Results from monté races (ridden races), which are not common in Sweden, were excluded for both breeds.

Racing barefoot was defined as racing with barefoot hind hooves (and shod or barefoot front hooves). Barefoot racing has not been allowed in winter (defined as a period ranging from December 1 to February 28) since 2015; therefore, all winter races were excluded when defining the proportion of barefoot races. Also, observations from the track condition “winter track” occurring outside of the winter season were removed because all horses raced shod in this track condition.

For SB, the horses were required to have at least five races as a 3-year-old. The total number of SB was 12,161, whereof 5,975 were females and 6,186 males. For CB, the horses were required to have at least five races as 3- and 4-year-olds, whereof at least one start as a 3-year-old. In total, 1,392 CB were included (593 females and 799 males).

### Risk factors

**Figure 1:**
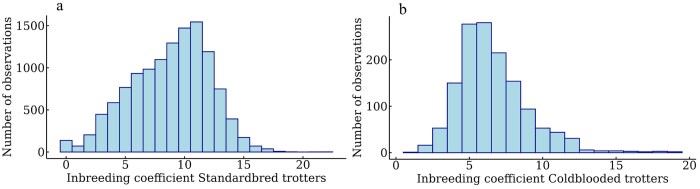
Distribution of inbreeding coefficient in the scale 0%–100% among the (a) Swedish Standardbred trotters and (b) Swedish-Norwegian Coldblooded trotters.

For both breeds, information about the inbreeding coefficient and the number of early starts, early earnings, record racing time per kilometer, and the proportion of barefoot races within the studied age span was included. Descriptive statistics of raw data before transformation are shown in Table 1. The inbreeding coefficients were estimated with all available pedigree information, which for SB included 305,044 animals and for CB, 118,239 animals. The inbreeding coefficients were estimated with the INBUPGF90 in the BLUPF90 suite of programs by Misztal et al. (2014). The distribution of inbreeding coefficients for SB and CB is shown in Figure 1. Pedigree completeness was analyzed with the CFC software (Sargolzaei et al. 2006), and the average number of discrete generation equivalents for SB and CB included in the study was 10.5 and 10.6 generations, respectively.

**Table 1:** Descriptive statistics of data before transformation, including mean, median, SD, minimum (min), and maximum (max) values for the number of early starts, early earnings, inbreeding coefficient, the proportion of barefoot races, and best racing time in Swedish Standardbred trotters (SB) and Swedish-Norwegian Coldblooded trotters (CB).

Breed	Group	Trait	Mean	Median	SD	Min	Max
**SB**	3-year-olds	Number of early starts^1^	9.8	9.0	3.4	5.0	30.0
		Number of early starts^2^	8.4	8.0	2.9	5.0	24.0
		Early earnings^3^	127,498	68,800	279,801	0.0	6,675,000
		Inbreeding coefficient	0.09	0.09	0.03	0.0	0.2
		Proportion of barefoot races	0.20	0.09	0.27	0.0	1.0
		Best racing time^4^	1,168	1,168	13	1,118	1,243
**CB**	3- and 4-year-olds	Number of early starts^1^	16.1	15.0	7.13	5.0	55.0
		Number of early starts^2^	13.7	13.0	6.36	5.0	43.0
		Early earnings^3^	121,584	72,250	158,260	0	1,844,000
		Inbreeding coefficient	0.07	0.06	0.02	0.013	0.19
		Proportion of barefoot races	0.05	0.00	0.12	0.000	1.0
		Best racing time^4^	1,313	1,312	32.0	1,235	1,445

1 Including all races

2 Excluding winter races

3 In SEK

4 Where eg 1,168 is 1 min 16 s and 8 tenths of a second

The factor proportion of barefoot races was, in SB, the relative frequency of barefoot races as a 3-year-old in races from March 1 to November 30. For CB, the proportion of barefoot races was summarized from both 3- and 4-year-old records for each horse. Swedish Standardbred trotters are allowed to start racing at the age of 2; therefore, information about whether the horse raced as a 2-year-old or not (regardless of time of the year) was included as a binary factor. Seventeen percent of the horses raced as 2-year-olds. This factor was not included in CB because they are not allowed to race as 2-year-olds.

**Figure 2:**
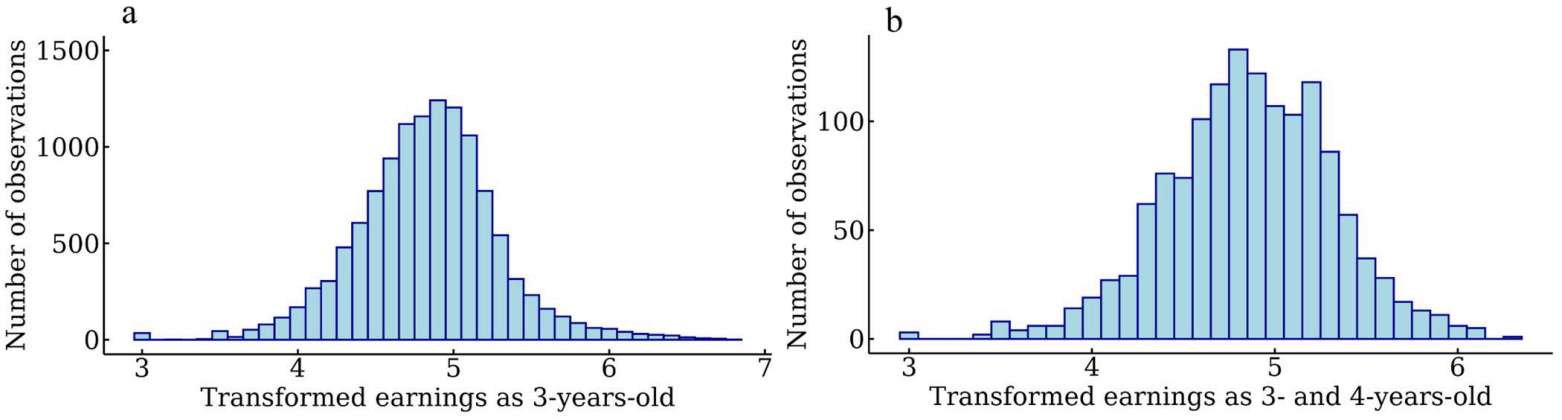
Distribution of transformed summarized early earnings in a) 3-year-old Swedish Standardbred trotters and b) 3- and 4-year-old Swedish-Norwegian Coldblooded trotters.

**Figure 3:**
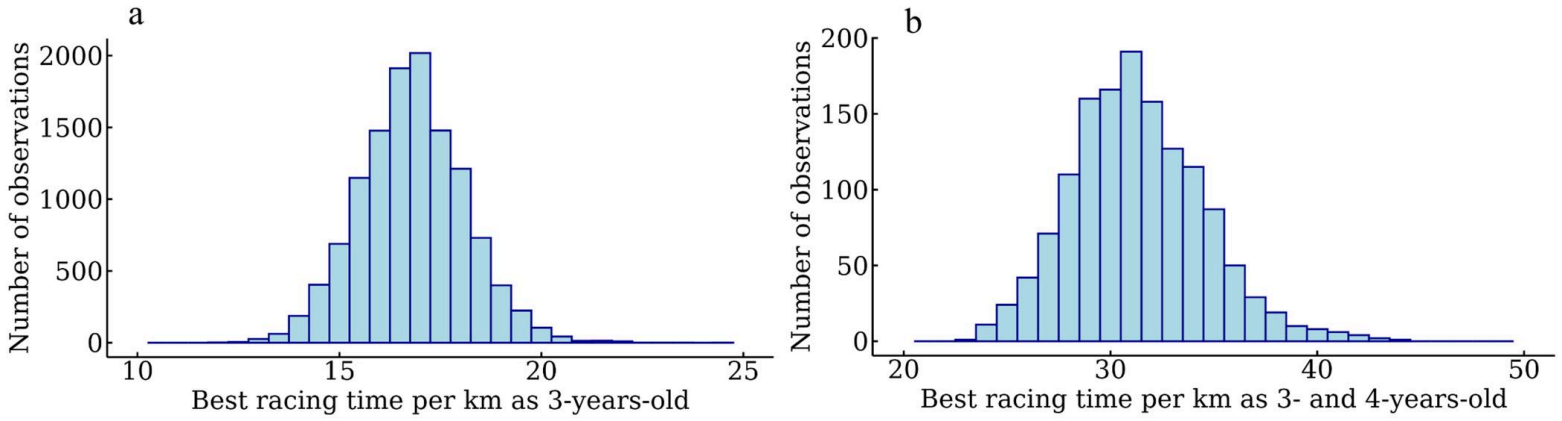
Distribution of the best racing time per kilometer among (a) 3-year-old Swedish Standardbred trotters and (b) Swedish-Norwegian Coldblooded trotters in the format of seconds over 1 min.

Including observations from all seasons, the number of early starts as a 3-year-old for SB, or 3- and 4-year-old for CB was summarized for all races. The number of starts from March 1 to November 30 was also summarized to calculate the proportion of barefoot races in this period. The summarized earnings (early earnings), including winter races as a 3-year-old for SB or as 3- and 4-year-old for CB were included as earnings in SEK + 1000, log-transformed to a base 10 scale for both breeds, following the standard procedure in the genetic evaluation for SB. The distribution of earnings after transformation is shown in Figure 2. The best racing time per kilometer had the raw data in the format of, eg 1,230, which is 1 min and 23 s. Two seconds were added to the results from autostart (start behind a car) following Árnason (2001). Because all horses had a best racing time per kilometer above 1 min and below 2 min, 1 min could be removed to have all times in the format of seconds over 1 min, as in the standard procedure in the genetic evaluation of SB and CB. In Figure 3, the trait distribution of best racing time per kilometer after transformation is shown.

### Trait definition and censoring

The career length was for both breeds defined as the number of days from the first race of the calendar year when the horse became 3-year-old until the horse ended the career or was censored as described below. Right censoring of horses in the dataset was applied to consider age- and sex-specific regulations for maximum age when the horses were allowed to race and to handle horses with an ongoing racing career at the time of the study. The Swedish Trotting Association has age restrictions, where for SB, restrictions are sex-specific and have changed over the years studied. In 2023 it was decided that mares born in 2012 and later have the same age restrictions as males, which are allowed to race from age 2 to 14 (Svensk Travsport 2025a). All horses that raced at the maximum age allowed according to the regulations at the specific time point were treated as right-censored because they may have been able to continue their careers if allowed. For SB included in the current study, the following age restrictions were applied: SB males born in 2006 and earlier were allowed to race until age 12, whereas for SB males born in 2007 and later, the maximum age was 14. For SB females born in 2011 and earlier, the age limit was 10, and for SB females born in 2012 and later, the age limit was 12 yr. Coldblooded trotters are allowed to compete from age 3 to 15 in both sexes (Svensk Travsport 2025a). As an example, SB males and females born in 2002 that were still racing at an age of 12 and 10 yr, respectively, were right-censored in this study. For CB, horses born between 2002 and 2006 that raced until age 15 were treated as right censored.

Additionally, all horses that raced within 2 yr from the last day of the data were treated as right censored in the analysis. A 2-yr period was chosen to ensure that horses had ended their careers and could be distinguished from those still racing or having a longer interruption (eg recovering from an injury). The suitability of this time span was confirmed by representatives of the trotting industry. A 1-yr cutoff period was argued to be too strict as it would not capture horses that were temporarily absent from racing. For SB, the last records were from December 18, 2022 and thus the cutoff date for censoring horses was 2 yr before that, ie horses still competing after December 18, 2020. As an example, all SB that had at least one start in the period December 18, 2020 to December 18, 2022 were treated as censored, while SB without racing results from this period were assumed to have ended their careers and thus not treated as censored. For CB, the data comprised records until December 29, 2021 and the cutoff date for censoring horses that were still competing was December 29, 2019. Censoring of horses due to age and sex-specific regulations, or due to an ongoing racing career, resulted in 30% and 39% of the horses being censored in SB and CB, respectively.

### Survival analysis

The data was fitted using the Cox proportional hazard model (Cox 1972; Therneau and Grambsch 2000) in R Studio with the Survival package (Therneau 2024). The time variable was career length in days, which, for both breeds, started from the first race of the calendar year when the horse turned three. To compare the rate at which horses in different groups (levels of fixed effects) end their career, the following Cox regression model was used:


$$h_{i}(t)=\;h_{0}(t)*\exp(X\beta ),$$

where $h_{i}(t)$ is the hazard for an individual at time t, $h_{0}(t)$ is the baseline hazard at time t, X is the vector of the covariates, and β is a vector of fixed effects as defined below. For the variable sex (female or male), both geldings and stallions were in the same group because there is no information on when the horses were gelded. Also, initial analysis of hazard ratios (HR) showed no difference between geldings and stallions. The birth years were divided into groups to distribute the horses evenly and ensure equal accuracy for all groups: 2002–2006, 2007–2010, 2011–2014, or 2015–2018 for SB and 2015–2017 for CB. Started as a 2-year-old or not was included as a binary trait (SB only). The number of early starts (also including races in the winter season, defined as ranging from December 1 to February 28) was divided into the following groups for SB to distribute the horses evenly: 5 to 10, 11 to 15, or 16 to 30 races. For CB, the number of early starts per group spanned from 5 to 10, 11 to 15, 16 to 20, or 21–55. The proportion of barefoot races was grouped into five classes for SB to distribute the horses evenly: ≤ 0.05, > 0.05 to ≤ 0.15, > 0.15 to ≤ 0.3, > 0.3 to ≤ 0.5, and > 0.5. For CB, the proportion of barefoot races was grouped into four classes: ≤ 0.05, > 0.05 to ≤ 0.15, > 0.15 to ≤ 0.3, and > 0.3. The transformed summarized early earnings were included as linear and quadratic regressions. Due to the non-linear shape of early earnings, adding a quadratic regression significantly improved the model. Interactions between the main effects of sex and early earnings, and sex and early earnings^2^ were also included in the model. The inbreeding coefficient on a scale from 0 to 1 was multiplied by 100 (corresponding to a percentage) and included as a linear regression in the model. The best racing time per kilometer in the format of seconds over one minute was also included as a linear regression.

The significance of the main effects in the model was tested with the chi-square test using the likelihood ratio test. Estimated coefficients and HR ie the hazard rate of the group of interest divided by the hazard rate of the reference group, including standard deviation, confidence intervals, and *P*-values were obtained for each effect and level from the Cox proportional hazard model. The proportional hazard assumption was checked by visual inspection of the scaled Schoenfeld residuals plotted against the transformed time, ensuring no pattern of the residuals with time (Schoenfeld 1982). From the Cox proportional hazard model, the Cox model-based survival curves were predicted with survfit in the Survival package (Therneau 2024) for the different levels of proportion of barefoot races, inbreeding, and best racing time stratified by sex. The survival function was defined as:


$$S_{i}(t)={\left[S_{0}(t)\right]}^{\exp(X\beta )},$$

where $S_{i}(t)$ is the survival of an individual at time t, $S_{0}(t)$ is the baseline survival function, X is the vector of the covariates, and β is a vector of fixed effects as defined above. Survival curves and median career length were estimated for all levels of proportion of barefoot races, for the first quartile, mean, and third quartile of the inbreeding coefficient. For best racing time (in seconds over one minute per kilometer), survival curves and median career length were estimated for 12 s, 17 s, and 24 s in SB, and in CB for 25 s, 31 s, and 40 s.

For illustrating the survival curves for the different levels of proportion of barefoot races, the other non-stratified factors in the model that were included as regressions were set to the average: the inbreeding coefficient was set to 9% in SB and 7% in CB, and the average racing time to 17 s in SB and 31 s in CB. For the survival curves and median career lengths for different inbreeding coefficients, the proportion of barefoot races was set to ≤ 0.05, and the best racing time to the average for SB and CB, respectively. For the survival curves and median career length of best racing time, the average inbreeding was used, and the level of the proportion of barefoot races was set to ≤ 0.05. For all stratified effects (proportion of barefoot races, inbreeding level, and best racing time), early earnings and early earnings^2^ were set to the mean value for SB and CB, respectively. For all curves, the year of birth was set to 2002–2006, and for SB, the not-started level as a 2-year-old was used. In both breeds, the number of early starts was set to 5–10. Survival curves were generated with ggsurvplot, and thereafter, median survival in days, including upper and lower 95% confidence intervals, was obtained for males and females separately.

## Results

### Survival analysis—coefficients and hazard ratios

**Figure 4:**
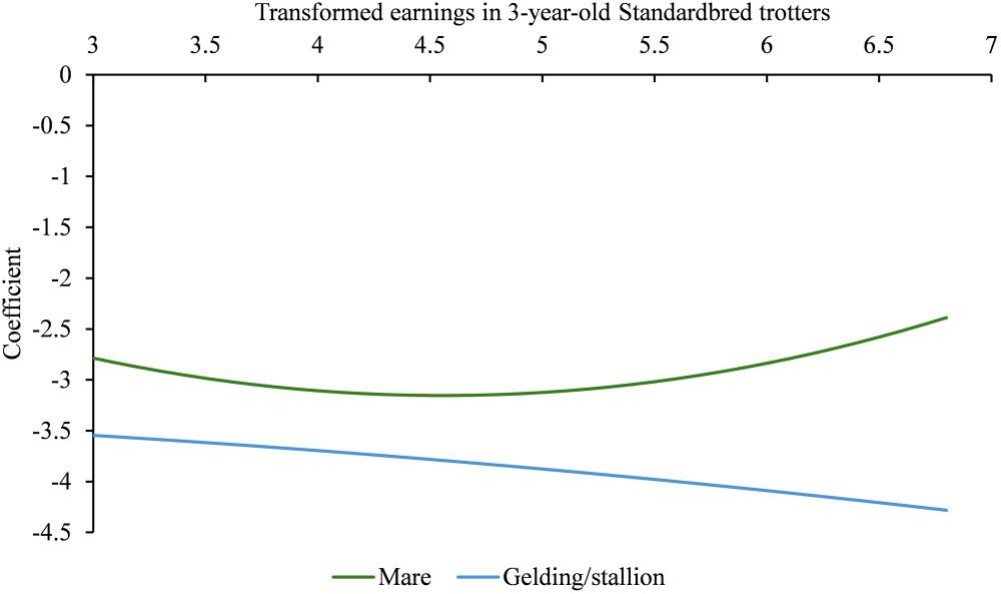
The effect on hazard for females (mare) and males (gelding/stallion) of log10 transformed early earnings in Swedish Standardbred trotters with at least five races as 3-year-olds.

***Standardbred trotters.*** For SB, results from the Cox model are shown in Table 2, including the significance level obtained from the likelihood ratio test with the chi-square test for the main effects. The two later birth year groups (2011 to 2014 and 2015 to 2018) were shown to have a significantly lower HR (HR < 1), indicating a lower risk of ending the career compared to the reference group (horses born in 2002 to 2006) at *P* < 0.001. Also, there was a significant interaction between sex and early earnings (*P* = 0.010) (shown in Figure 4). With high prize money, the trend line for females had a positive non-linear slope, ie higher early earnings were associated with an increased HR and higher risk of ending the career. For males, the trend line had a negative slope, meaning that higher early earnings were associated with a lower HR. However, across the entire earnings range, males had a lower probability of ending the career compared with females.

**Table 2:** Number of horses, survival coefficients with SE, hazard ratio, and 95% confidence interval for hazard ratios in Swedish Standardbred trotters with at least five races as 3-year-olds. The level of significance for the main effects is indicated with *^,^  **^,^  ***.

Factor	N	Coefficient	SE coefficient	Hazard ratio	Lower CI 95%	Upper CI 95%	*P*-value
Year of birth***							
**2002–2006**	3,029	0		1			
**2007–2010**	2,973	−0.04	0.027	0.96	0.92	1.02	0.173
**2011–2014**	3,096	−0.25	0.030	0.78	0.73	0.82	<0.001
**2015–2018**	3,063	−1.02	0.050	0.36	0.33	0.40	<0.001
Sex**							
**Female**	5,975	0		1			
**Male**	6,186	−3.29	1.262	0.04	0.00	0.444	0.009
***Started as 2 years old***							
**No**	9,690	0		1			
**Yes**	2,471	0.04	0.028	1.04	0.99	1.099	0.142
Number of early starts***							
**5–10**	7,641	0		1			
**11–15**	3,774	−0.11	0.026	0.90	0.85	0.948	<0.001
**16–30**	746	−0.30	0.049	0.74	0.67	0.813	<0.001
Early earnings***	12,161	−1.38	0.362	0.25	0.12	0.509	<0.001
Early earnings^2^***	12,161	0.15	0.037	1.16	1.08	1.252	<0.001
Inbreeding coefficient***	12,161	0.02	0.003	1.02	1.01	1.025	<0.001
Proportion of barefoot races***							
**≤ 0.05**	5,784	0		1			
**> 0.05 & ≤ 0.15**	1,317	0.08	0.037	1.09	1.01	1.17	0.026
**> 0.15 & ≤ 0.3**	1,695	0.10	0.033	1.10	1.03	1.18	0.004
**> 0.3 & ≤ 0.5**	1,637	0.13	0.034	1.14	1.07	1.22	<0.001
**> 0.5**	1,728	0.13	0.033	1.14	1.07	1.22	<0.001
***Best racing time***	12,161	−0.01	0.013	0.99	0.96	1.02	0.406
SexMale: Early earnings**	12,161	1.35	0.525	3.84	1.37	10.737	0.010
SexMale: Early earnings^2^**	12,161	−0.17	0.054	0.85	0.76	0.941	0.002

*0.01 < *P* ≤ 0.05; **0.001 < *P* ≤ 0.01; ***P ≤ 0.001.

In SB, neither starting as a 2-year-old nor the best racing time as a 3-year-old had any significant effect on the HR (*P* = 0.142 and *P* = 0.406, respectively). The level of inbreeding had a slightly negative effect on the HR, estimated at 1.025 at *P* < 0.001. This means that for each percentage unit increase of the inbreeding coefficient, the hazard of ending the career at a specific time point increases by 2.5%. A higher number of starts as a 3-year-old was associated with a lower HR (*P* < 0.001), and horses in the two groups with 11 races or more had an HR estimated at 0.90 and 0.74, respectively, compared to the reference group that started in 5 to 10 races. This means that racing more than 11 races as a 3-year-old decreased the hazard of ending the career compared to those starting only in a few races.

In SB, horses in all groups that raced more than 5% barefoot had a significantly higher HR than those that raced barefoot the least (≤ 5%). The HR increased with higher proportions of barefoot races, except between the two groups with the highest proportion of barefoot races (> 0.3 to ≤ 0.5 and > 0.5), where the HR was 1.14 for both. An HR of 1.14 means that horses that raced more than 30% barefoot as 3-year-olds had a 14% higher hazard rate of ending their career at a given time point than those racing up to 5% barefoot.

***Coldblooded trotters.*** For CB, results from the Cox analysis, including the significance levels obtained from the likelihood ratio test with the chi-square test for the main effects, are shown in Table 3. Horses born in the last birth year group (2015–2017) had a significantly lower HR (0.22) at *P* < 0.001 than those in the reference group born in 2002–2006, ie a lower hazard of ending the career at a given time point. For CB, the main effects and the interactions between sex and early earnings were not significant. Horses starting in 16 races or more (as a 3- and 4-year-old) had a significantly lower HR (< 1) compared to those starting in 5 to 10 races (*P* = 0.001). Horses in the group that started the most races (20 to 55 races) had an HR of 0.59 (*P* < 0.001), which means that the hazard of ending the career at a given time point is reduced by 41% compared to the group with 5 to 10 races. The inbreeding coefficient had no significant effect on the hazard for this breed. However, unlike SB, the best racing time as a 3- and 4-year-old was highly significant (*P* < 0.001) with an HR of 1.12. This means that for each second slower the horses raced, the hazard of ending the career at a given time point increased by 12%.

**Table 3:** Number of horses, estimated coefficients with SE, hazard ratio and 95% confidence interval (CI) for hazard ratios in 3- and 4-year-old Swedish-Norwegian Coldblooded trotters. The level of significance for the main effects is indicated with *^,^  **^,^  ***.

Effect	N	Coefficient	SE coefficient	Hazard ratio	Lower CI 95%	Upper CI 95%	*P*-value
Year of birth***							
**2002–2006**	330	0		1			
**2007–2010**	389	−0.06	0.081	0.94	0.80	1.11	0.462
**2011–2014**	363	−0.17	0.099	0.85	0.70	1.03	0.089
**2015–2017**	310	−1.52	0.248	0.22	0.13	0.36	<0.001
***Sex***							
**Female**	593	0		1			
**Male**	799	2.64	4.743	13.94	0.00	151858.10	0.578
Number of early starts***							
**5–10**	338	0		1			
**11–15**	400	0.05	0.100	1.05	0.86	1.28	0.623
**16–20**	296	−0.40	0.125	0.67	0.52	0.86	0.001
**20–55**	358	−0.52	0.136	0.59	0.45	0.77	<0.001
***Early earnings***	1,392	0.49	1.556	1.63	0.08	34.49	0.753
***Early earnings^2^***	1,392	0.02	0.165	1.02	0.74	1.41	0.911
***Inbreeding coefficient***	1,392	0.00	0.016	1.00	0.97	1.03	0.987
Proportion of barefoot races**							
**≤ 0.05**	1,033	0		1			
** > 0.05 & ≤ 0.15**	188	0.20	0.102	1.22	1.00	1.50	0.047
** > 0.15 & ≤ 0.3**	104	0.21	0.133	1.24	0.95	1.61	0.113
** > 0.3**	67	0.51	0.168	1.67	1.20	2.33	0.002
Best racing time***	1,392	0.11	0.025	1.12	1.07	1.18	<0.001
***SexMale: Early earnings***	1,392	−1.00	2.015	0.37	0.01	19.08	0.619
***SexMale: Early earnings^2^***	1,392	0.07	0.213	1.08	0.71	1.64	0.729

* 0.01 < *P* ≤ 0.05; **0.001 < *P* ≤ 0.01; *** *P* ≤ 0.001.

For the proportion of barefoot races, 1,033 horses out of 1,392 started up to 5% of the races barefoot, which led to few observations in the remaining three groups. Horses that started more than 5% and up to 15% of the races barefoot had an HR of 1.22, which was significantly different from those that started 5% of the races or less barefoot (*P* = 0.047). The HR for the group of horses that started more than 15% and up to 30% of the races barefoot was 1.24, which was not significantly different from the group with 5% of the races or less races barefoot (*P* = 0.113). For the group of horses that raced more than 30% barefoot (with an average of 50%), the HR was 1.67, which was significantly higher than the group that raced 5% or less barefoot (*P* = 0.002). A HR of 1.67 means that the hazard of ending the career at a given time point increased by 67% compared to the group with the lowest proportion of barefoot races.

### Survival curves and median survival time

**Figure 5:**
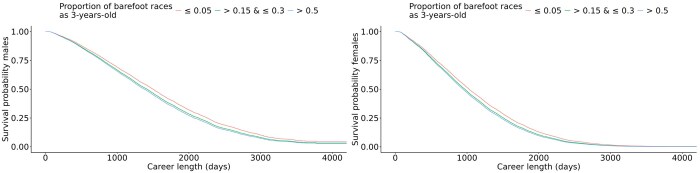
Survival curves for proportion of barefoot races for the levels ≤ 0.05, > 0.15 & ≤ 0.3, and > 0.5 in 3-year-old Standardbred trotters. Males are displayed on the left and females on the right.

***Standardbred trotters.*** Survival curves for different levels of proportion of barefoot races, separated for males and females in SB are shown in Figure 5. For both sexes, the groups with the lowest proportion of barefoot races had the longest career length. Also, the group with the highest proportion of barefoot races had the shortest career length. For females, the probability of a continued career reached zero at approximately 3,000 d, and for males, the probability of a continued career reached zero around 3,500 d.

The median career length for males in the reference group, racing 5% or less barefoot as 3-year-old was 1,501 d (4.1 yr) (Table 4). The group of males that started more than 50% of the races barefoot had a shortening of their careers by 123 d compared to the reference group. For females, the reference group had a median career length of 1,030 d (2.8 yr). Females that raced more than 50% barefoot had a shortening of the career length by 85 d compared to the reference group.

**Table 4:** Median survival time in days for different levels of the proportion of barefoot races and inbreeding for Standardbred trotters with at least 5 races as 3-years-old.

Trait	Sex	Level	Median survival time (days)	Diff days +/−	Lower CI 95%	Upper CI 95%
**Proportion of barefoot races**	Male	≤ 0.05	1,501		1,411	1,601
		> 0.05 & ≤ 0.15	1,429	−72	1,317	1,539
		> 0.15 & ≤ 0.3	1,415	−86	1,308	1,524
		> 0.3 & ≤ 0.5	1,381	−120	1,283	1,497
		> 0.5	1,378	−123	1,282	1,490
	Female	≤ 0.05	1,030		968	1,111
		> 0.05 & ≤ 0.15	979	−51	904	1,060
		> 0.15 & ≤ 0.3	971	−59	898	1,049
		> 0.3 & ≤ 0.5	947	−83	879	1,026
		> 0.5	945	−85	878	1,023
**Inbreeding Level**	Male	6%	1,545		1,458	1,653
		9%	1,501	−44	1,411	1,601
		11%	1,468	−77	1,373	1,570
	Female	6%	1,065		1,002	1,155
		9%	1,030	−35	968	1,111
		11%	1,009	−56	941	1,085

**Figure 6:**
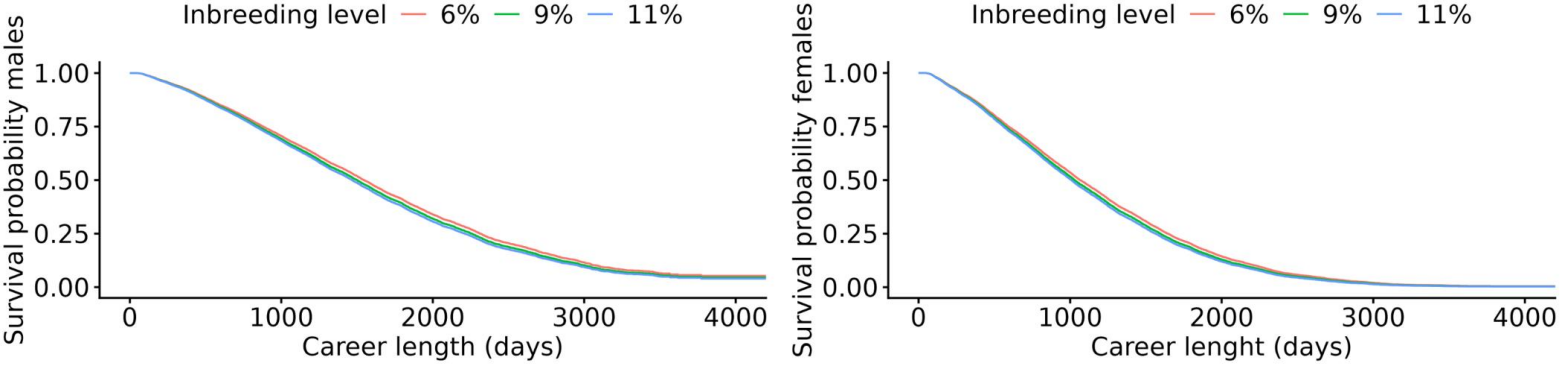
Survival curves for the first quartile (6%), mean (9%), and third quartile (11%) of inbreeding in the dataset with 3-year-old Standardbred trotters. Males are shown on the left and females on the right.

In Figure 6, three different survival curves were fitted, representing the first quartile (6%), mean (9%), and third quartile (11%) of inbreeding in SB. Although the effect of inbreeding level was significant, there were small differences in survival length being inbred at a level of 6, 9, or 11% as shown in Table 4. Having an inbreeding coefficient of 6% gave a median career length in males of 1,545 d (4.2 yr). An inbreeding coefficient of 11% reduced the career length by 77 d. In females, having an inbreeding coefficient of 6% gave a median career length of 1,065 d (2.9 yr). An inbreeding coefficient of 11% reduced the career length by 56 d compared to the 6% level.

**Figure 7:**
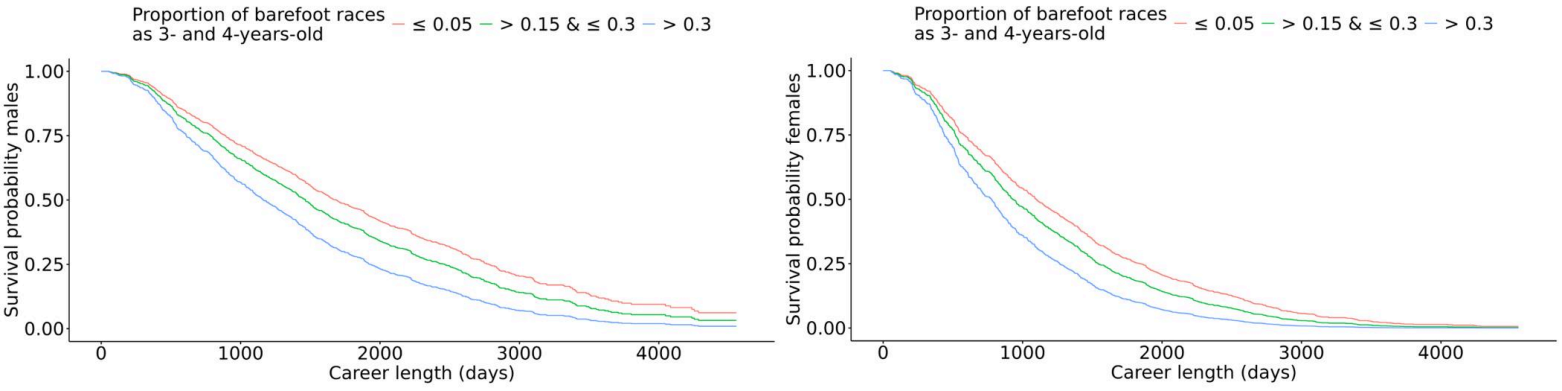
Survival curves for proportion of barefoot races in Swedish-Norwegian Coldblooded trotters for the levels ≤ 0.05, > 0.15 & ≤ 0.3, > 0.3. Males are displayed on the left and females on the right.

***Swedish-Norwegian Coldblooded trotters.*** The survival curves for three different levels of the proportion of barefoot races in 3- and 4-year-olds CB are shown in Figure 7, with the corresponding median career length for all levels in Table 5. The group with the highest proportion of barefoot races, more than 30% (50% on average), had the shortest career length for both males and females. For males in the reference group that raced 5% or less of the races barefoot as 3- and 4-year-olds, the median career length was 1,688 d (4.6 yr). For males that raced more than 30% barefoot, the career length was reduced by 523 d compared to the reference group. For females in the reference group, the median career length was 1,094 d (3 yr). Females racing more than 30% barefoot had a shortening of the career length by 316 d.

**Table 5:** Median survival time in days, difference in days from the reference level, lower and upper confidence intervals for Swedish-Norwegian Coldblooded trotters for different levels of proportion of barefoot races and best racing time.

Trait	Sex	Level	Median survival time (days)	Diff days +/-	Lower CI 95%	Upper CI 95%
**Proportion of barefoot races as 3- and 4-years-old**	Male	≤ 0.05	1,688		1,350	2,500
		> 0.05 & ≤ 0.15	1,466	−222	1,119	2,225
		> 0.15 & ≤ 0.3	1,464	−224	1,102	2,285
		> 0.3	1,165	−523	855	1,932
	Female	≤ 0.05	1,094		843	1,650
		> 0.05 & ≤ 0.15	941	−153	711	1,502
		> 0.15 & ≤ 0.3	927	−167	700	1,542
		> 0.3	778	−316	547	1,309
**Best racing time as 3- and 4-years-old**	Male	25 s	2,758		2,110	4,254
		31 s	1,688	−1070	1,350	2,500
		40 s	811	−1947	557	1,603
	Female	25 s	1,813		1,356	2,973
		31 s	1,094	−719	843	1,650
		40 s	535	−1278	423	1,068

**Figure 8:**
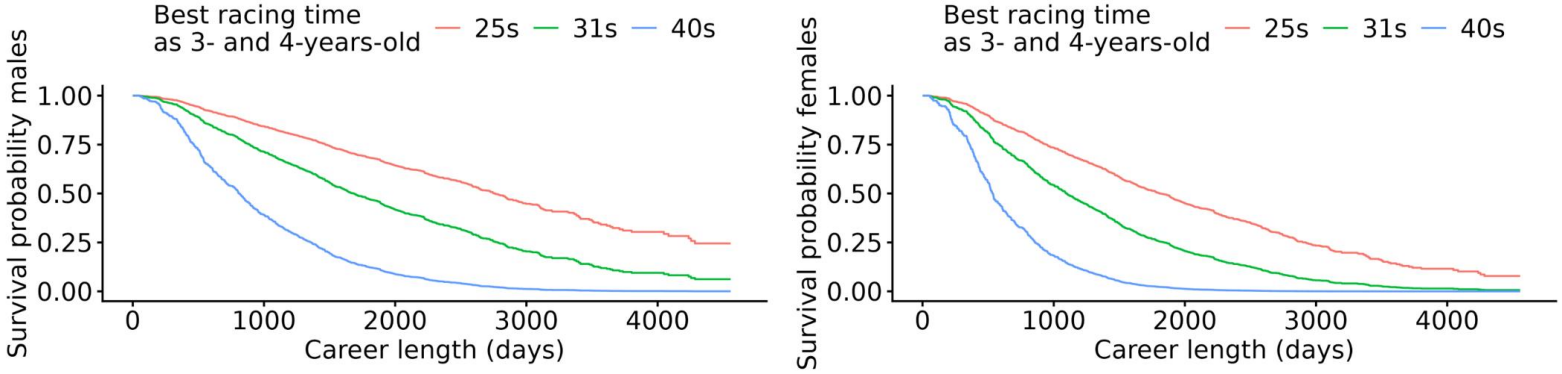
Survival curves for best racing time in the format of seconds over one minute for 25 s, 31 s, and 40 s per kilometer in 3- and 4-year-old Swedish-Norwegian Coldblooded trotters. Males are displayed on the left and females on the right.

The best racing time in CB as a 3- and 4-year-old was highly significant for the career length. In Figure 8, survival curves from a best racing time of 1 min and 25 s, the average best racing time of 1 min and 31 s, and at 1 min and 40 s are displayed in seconds over 1 min. The corresponding median career length of these racing times is shown in Table 5.

As seen in Figure 8, a clear distinction of the different racing times was seen, where being fast as young seems to be very important for the career length in CB. For males, having a racing time of 1 min and 25 s gave a median career length of 2,758 d (7.6 yr), while racing at 1 min and 40 s gave a reduction in career length by 1,947 d (5.3 yr) compared to the former. Females racing at 1 min and 25 s had a median career length of 1,813 d (5 yr), whereas those racing at 1 min and 40 s had a 1,278 d (3.5 yr) shorter career length compared to those racing at 1 min and 25 s.

## Discussion

There is a general perception that barefoot racing may have a negative impact on animal welfare because it causes excessive wear and tear of the hooves, which potentially could have a negative effect on the long-term durability of the horses. To race without shoes protecting the hoof, ie racing barefoot, especially in young trotters, is a debated topic in Sweden but also in other European countries. However, large-scale studies of the impact of racing barefoot on career length have been missing. This study shed light on the hazard of ending the career for different levels of proportion of barefoot races as a young horse.

### Data material and trait definitions

By using repeated observations from all trotting races in Sweden for SB and CB, it was possible to estimate the total career length in days, in contrast to a previous study of career length in SB that used yearly summarized data (Árnason 2006). The intention of studying the effect on barefoot racing limited the data to horses that raced during the years when shoeing information was available. Especially for CB races, which constitute only 10% of the 8,000 races that are arranged annually in Sweden (Svensk Travsport 2025d), this led to a small dataset and the results for CB must therefore be interpreted with caution. Also, the strict editing of only keeping horses with five races or more in the non-winter season for SB and CB could also have made the results less representative of the full populations. However, this requirement was based on that racing barefoot in 100% of the races, when very few races were performed, would not reflect the hoof’s ability to tolerate barefoot racing repeatedly compared to racing barefoot repeatedly across 5, 10, or more races. Excluding foreign horses was necessary to assess the career length as accurately as possible and to properly define the different risk factors.

Further, using survival analysis, horses that had not finished their racing careers at the end of the time period that the datasets covered could still be included and contribute to the analysis, although treated as censored. Especially for CB, many horses were censored (39%), and horses born from 2002 to 2006 were those who contributed with their full career length in the data (up to age 15). In SB, the proportion of horses censored was somewhat lower (30%). However, the birth year group was shown to have a significant effect on the HR, with lower HR (< 1) for more recent birth years. A similar pattern was shown for SB in the study by Árnason (2006), including data from races up to 2005, where the hazard declined from −0.2 for males born 1976 to −0.8 for males born 2001 and from 0.8 to −0.6 for females in the same time period. Whereas we cannot rule out an effect of the proportion of censored horses in the different birth cohorts, there may also be favorable influences from improved management and training, refined regulations to enhance animal welfare, and breeding of horses better suited to the sport.

Early performance, ie earnings, best racing time, and number of early starts, was included in the models to separate strategic and voluntary endings of the careers from those of an involuntary type. Although we do not have information on why the individual horses ended their careers, those of the voluntary type could be to use a successful mare for breeding or to end the career of a less talented horse due to insufficient earnings, and those of the involuntary type could, for example, be due to injuries. For SB, early earnings were shown to have a significant effect on the career length, with higher early earnings being favorable for the career length, whereas for CB, which also included the 4-year-old season, it was not significant. Árnason (2006) has previously studied the correlation between early racing performance in SB and different stayability indices. The stayability index reflected the hazard of ending the career based on survival analysis corrected for the interaction of sex, birth year, and inbreeding level. Results showed that the correlation between the different stayability indices and the total index for the breeding value for stallions for racing performance as a 3- to 5-year-old was favorable. Also in Thoroughbreds, early earnings have been shown to have a significant effect on career length, with lower HR (<1) ie higher log earnings in the first racing season were favorable for the career length (Sobczynska 2007).

For SB in our study, it was shown that high early earnings for females (mares) increased the hazard, while for males, it decreased the hazard. Mares have an alternative value in breeding, while geldings (ie most males in the data) do not, and therefore continue to race. Hence, the retirement of the most successful young mares was likely a voluntary decision. In initial analysis, geldings and stallions were divided into separate groups, but the survival probability over time did not differ. Also, the data contained no information about at what age males were gelded. Therefore, they were grouped together in the final model used. The effect of sex on career length in SB born 1976 to 2001 also showed a higher HR (>1) for females (mares) compared to males (geldings and stallions), according to Árnason (2006). That mares commonly have shorter careers in the sport due to their value in breeding has also been shown in riding horses (Ducro et al. 2009; Ricard and Blouin 2011).

In the current study, all horses were required to have started their career as 3-year-olds. However, in SB, horses are allowed to start racing already as 2-year-olds, and this was corrected for by adding a binary effect of having raced as 2-year-olds or not in the model. The effect of racing as a 2-year-old was not significant for career length of SB in this study, however. Our results are not in line with the results from Velie et al. (2019) that showed that starting the career early in CB was positive for career longevity. In that previous study, there was possibly an autocorrelation between a longer career and an early start of the racing career because this enabled the horses to have more years to race. In our study, career length was measured from 3 yr of age for all horses.

A higher number of starts as a young horse was shown to be associated with a HR <1 for all datasets compared to the reference groups, which had the fewest races. This indicates that the intensity of racing in young trotters in Sweden is not harmful to their career length, and it implies that the trainers have a long-term perspective when adapting the amount of racing for the young horse. It is also likely that more talented horses in good health are raced more when young. Also in Italian Standardbreds, Bertuglia et al. (2014) showed that the incidence rate of obtaining musculoskeletal injuries in a 4-year-period decreased with a higher average number of starts per year. Horses in all groups racing more than four races per year had a significantly lower risk ratio of obtaining injuries compared to the reference group that raced between 0 and 4 races per year on average. However, in Thoroughbreds, a higher number of starts in the first racing season was found to have a negative effect on career length, where the hazard of ending the career at a given time point increased with 4% per start (Sobczynska 2007). Bertuglia et al. (2014) also found a higher incidence rate of musculoskeletal injuries for horses with a slower best racing time, while in pacers, Rouette et al. (2021) found that a higher speed was associated with an increased risk of injuries. In the current study, a fast record racing time per kilometer was associated with a longer racing career in CB only.

A higher inbreeding level was shown to have a negative effect on career length in SB but not in CB. For the horses included in this study, the average inbreeding coefficient was higher for SB (0.09) compared to CB (0.06). However, the mean inbreeding level for the subset of CB included in this study was lower than what has previously been estimated for all CB born in 2021, where it was estimated at 0.08 (Berglund et al. 2024). The distribution of inbreeding coefficients seems somewhat skewed, where for SB, there were more horses with higher inbreeding coefficients, and for CB, there were more horses with lower inbreeding coefficients. While for SB, our results are in line with Árnason (2006), who also found a negative effect of having a higher inbreeding coefficient on career length, the results for CB may not represent the full population due to the few horses included in our study.

Solé et al. (2017) found that having a more experienced rider lowered the risk of ending the career in horses of the Spanish horse breed Pura Raza Español. In the current study, it was not possible to correct the effect of the trainer and driver because trotting horses commonly change trainers and drivers during their careers. However, the trainer of trotting horses is known to have an impact on the performance of the horse (Berglund et al. 2024).

### The effect of racing barefoot as a young horse

The results from this study showed that a higher proportion of barefoot races at a young age were associated with a shorter career in both breeds. Between the years 2005 and 2022, the percentage of 3-year-old SB racing fully barefoot, which is less common than racing with barefoot hind hooves, has ranged from 7.5% up to 13.2% (Svensk Travsport 2023b). In the current study, where racing barefoot was defined as racing with barefoot hind hooves and shod or barefoot front hooves, the overall mean for the same period was 20%. Previously, Berglund et al. (2025a) showed that on average, 29% of the starts in 3- to 10-year-old SB were with barefoot hind hooves, but the probability of racing barefoot also increased with age. The requirement in the number of early starts (at least five) would probably lead to a higher probability of at least once making an attempt to race barefoot. Whereas in the full dataset, trainers of horses that only start in one or a few races will probably not attempt to race the horses barefoot that early in their career. In CB, the horses on average raced barefoot hind 10% of the races as 3- to 10-year-olds (Berglund et al. 2025a), compared to the mean value of 5% in the current study for 3- and 4-year-olds. Also for CB, the probability of racing barefoot increased with age (Berglund et al. 2025a).

In SB, already when racing over 5% of the races barefoot, this was associated with an increased HR (> 1) compared to racing 5% or less barefoot. A higher proportion of barefoot races as young was associated with an increased HR. However, the difference in career length between the groups with the highest and lowest proportion of barefoot races was only 123 and 85 d in SB males and females, respectively. It was only for CB that a large difference was seen in the actual impact on career length, where the group that raced more than 30% of the races barefoot had 523 d (males) and 316 d (females) shorter career lengths compared to the reference group (5% or less barefoot). In CB, few horses raced more than 5% barefoot and racing more than 15% up to 30% was not significantly different from the reference level. However, the other levels (more than 5 but less than 15% and also 30% or more) were significantly different from the reference level and verified the negative effect found for SB.

### The physical effect of barefoot racing on racing durability

The effect of barefoot racing on the risk of injuries in trotters has been shown to differ between studies. Bertuglia et al. (2014) did not find any significant difference in incidence rate of musculoskeletal injuries between trotters racing shod versus barefoot. However, racing barefoot hind has been shown to increase the risk of injuries on the front legs caused by interference with the hind hooves (Dabbene et al. 2018). The authors also found that breaking over to gallop increased the risk of interference injuries. The link between racing barefoot hind and an increased risk of breaking over to gallop has previously been shown by Solé et al. (2020) in SB.

While the shoe protects the hoof from wear and tear, it will also affect the hoof mechanism, ie the elastic properties of the hoof and how these change when the horse moves and the hoof deforms when in contact with the ground surface. Heel expansion in the stance phase (ie when the hoof is in contact with the ground surface) increases by speed, but also when the hoof is barefoot compared to shod (Roepstorff et al. 2001; Brunsting et al. 2019). Also, if the hooves are shod, additional factors can influence the hoof mechanism, such as the placement of nails (Dahl et al. 2023). Using alternative methods such as attaching the shoe with glue has also been shown to influence hoof expansion, and thereby, the shock-absorbing properties of hoof (Yoshihara et al. 2010).

The force induced from the ground to the limb when the hoof meets the ground in the stance phase is often referred to as the ground reaction force (Wilson and Weller 2011). When unshod in trot, the weight of the hoof is reduced, and the hoof has a lower velocity when landing; this leads to a reduced vertical force from the hoof to the ground compared to when shod (Roepstorff et al. 1999).

The reason some horses have hooves that tolerate excessive wear and tear from barefoot racing and others suffer from damage to the hoof if raced barefoot could partly be explained by the composition of chemicals in the hoof wall of the hind hooves, pointing at differences in hoof durability (Spörndly-Nees et al. 2023). That study showed that horses reported to tolerate racing barefoot repeatedly without excessive wear and tear of the hooves had higher concentrations of arginine, proline, and cysteine in the hoof wall than those reported not to. These amino acids are all involved in protein synthesis, and cysteine is particularly important for keratin structure, which has an impact on hoof strength (Zhang et al. 2026). Further, the authors reported higher concentrations of sulfur in the hoof wall of horses reported to tolerate the wear from barefoot racing. A higher concentration of sulfur in the hoof wall has previously been linked to an increased tensile strength (Ley et al. 1998).

The hoof wall is connected to the distal phalanx via the primary epidermal laminae (PEL), which helps to distribute load and manage the vertical ground reaction forces. This tissue undergoes changes as the horse ages, adapting to carry the mass of the horse (Bowker 2003). It has been shown that the PEL differs in density between fetuses of feral horses and Thoroughbreds, where the feral fetus had a higher density of PEL (Hampson et al. 2011). This indicates genetic differences in terms of the development of this tissue as a fetus.

As previously mentioned, Schwochow et al. (2025) reported differences in gene expression in the coronary band of hind hooves between trotters able to sustain barefoot racing without hoof damage and those that could not. These differences were linked to biological mechanisms playing an important role in hoof durability and, consequently, in the ability to race barefoot. However, studies on hoof tissue require biopsies, which cannot be collected from living horses. For breeding purposes, it would be advantageous to measure traits early in life that reflect a trotter’s ability to withstand barefoot racing without excessive wear of the hooves. Therefore, routinely collected indirect phenotypic measures that can be associated with functional properties of the hoof would be preferable.

The ability to race barefoot hind in trotters, defined as the proportion of barefoot races, but also the probability to race barefoot in a specific race, has been shown to partly be explained by the genetic background of the horses, with heritability estimates in the range 0.07–0.28 (Berglund et al. 2025a). Also, genetic correlations between the proportion of barefoot races or barefoot status with performance traits included in the genetic evaluation of Swedish trotters (eg best racing time, earnings, and placings) were favorable (Berglund et al. 2025b). Although previous studies show potential to include a trait that measures the ability to race barefoot in the genetic evaluation, which may indirectly be used to improve aspects of hoof quality and potentially also balance, the results from the current study suggest a negative phenotypic effect of barefoot racing as a young horse on career longevity.

Racing barefoot hind is considered to primarily reflect the hoof’s ability to withstand wear and tear (Solé et al. 2020; Spörndly-Nees et al. 2023). Racing barefoot on the front hooves has been shown to increase the risk of breaking over to gallop, which trainers have suggested may be related to balance in the gait rather than wear of the hooves (Solé et al. 2020). This indicates that ability to race barefoot in hind versus in front hooves may have partly different biological explanations that warrant further investigation.

### Prevalence of injuries in trotting horses

The results in this study showed an overall negative effect of barefoot racing in young Swedish trotters on their career length. However, the actual reason why horses end their careers is unknown. Injuries are one of the plausible involuntary reasons for ending the racing career. Data obtained from surveys of veterinarians and trainers, together with the trainers’ own medical history of the horses, suggest that orthopedic problems are the most frequent type of injury (Holmquist 2023), but injuries to the hoof capsule were not specifically reported. The same author found that inflammation in the carpal joint was the most reported health issue, followed by inflammation to stifle joints, fetlocks, and coffin joints in the forelegs. Carpal joint lameness was shown to be more frequently observed in 4 years and younger trotters compared to older (Bertuglia et al. 2014). Vigre et al. (2002) also found an effect of age on the risk of lameness in trotters, where 3- and 4-year-olds had a higher HR compared to older horses. A study by Rouette et al. (2021), including both trotters and pacers, showed that most injuries occurred during training (53%), whereas 41% occurred in a race. It should be noted that the few trotters in their study (22 out of 153 horses) did not receive injuries, however.

### Implications

While our results demonstrate an increased HR with a larger proportion of barefoot races in 3-year-old SB, and also in 3- and 4-year-old CB, the actual difference in career length was small in SB. If the Swedish Trotting Association were to consider these results in favor of banning barefoot racing in 3-year-olds, Swedish races would probably have to shift from mixed to only age-specific races. In other European countries, such as France and Germany, barefoot racing is only allowed for 4-year-old trotters and older (The European Trotting Union 2024; The European Trotting Union 2025a), whereas in, eg Italy and Finland, the horses are allowed to race barefoot from 3 yr of age (The European Trotting Union 2025b; The European Trotting Union 2025c). In Finland, the Finnhorse, a horse of coldblooded type similar to CB, is allowed to race barefoot from 4 yr of age. Different regulations for the two breeds, as already implemented in Finland, seem reasonable according to our findings. Again, the small number of horses included for CB implies that the results should be interpreted with some care, however. Also, if regulations regarding barefoot racing change, this may have a negative effect on the potential of including this trait in the genetic evaluation of Swedish trotters to indirectly improve hoof quality and balance.

## Conclusions

To the best of our knowledge, this study provides the first results on the effect of barefoot racing in young trotters on their career length. We can conclude that starting in many races at a young age had a favorable effect on career length, while a higher proportion of barefoot races at a young age was shown to have a negative effect on career length. However, the reduction in the number of race days was relatively small for SB. For CB, the results were less clear due to fewer observations but verified the negative effect on career length with an increased proportion of barefoot races at a young age. For both SB and CB, further studies to investigate the actual reason for the horses ending their careers are recommended to increase the knowledge on why a higher proportion of barefoot races at a young age could lead to a shorter career length.

## Abbreviations

CB: Swedish-Norwegian Coldblooded trotter
HR: hazard ratio
PEL: primary epidermal laminae
SB: Swedish Standardbred trotter

## References

[skag127-B1] Aho E. 2025. Genetic parameters of earnings, best race time, and career length in standardbred trotters. Department of Agricultural Sciences. University of Helsinki. http://hdl.handle.net/10138/596627

[skag127-B3] Árnason T. 2001. Trends and asymptotic limits for racing speed in standardbred trotters. Livest Prod Sci. 72:135–145. 10.1016/S0301-6226(01)00274-3

[skag127-B4] Árnason T. 2006. Survival analysis of the length of competition life of Standardbred trotters in Sweden. The 57th annual meeting of the European association for animal production. p. 17–2.

[skag127-B5] Berglund P et al 2025a. The ability to race barefoot is a heritable trait in Standardbred and Coldblooded trotters. Genet Sel Evol. 57:8. 10.1186/s12711-025-00958-2 40000964 PMC11863800

[skag127-B6] Berglund P , Andonov S, Strandberg E, Eriksson S. 2024. Should performance at different race lengths be treated as genetically distinct traits in Coldblooded trotters? J Anim Breed Genet. 141:220–234. 10.1111/jbg.12837 38009381

[skag127-B7] Berglund P , Andonov S, Strandberg E, Eriksson S. 2025b. Better hoof, better horse - genetic correlations between ability to race barefoot and performance in Swedish trotting horses. Animal. 19:101664. 10.1016/j.animal.2025.101664 41108946

[skag127-B8] Bertuglia A , Bullone M, Rossotto F, Gasparini M. 2014. Epidemiology of musculoskeletal injuries in a population of harness Standardbred racehorses in training. BMC Vet Res. 10:11–10. 10.1186/1746-6148-10-11 24410888 PMC3922780

[skag127-B9] Boorman S et al 2021. Influence of osteochondrosis on the longevity and racing performance of standardbred trotters and pacers. Vet Surg. 50:507–516. 10.1111/vsu.13568c 33460472

[skag127-B10] Bowker RM. 2003. The growth and adaptive capabilities of the hoof wall and sole: functional changes in response to stress. The 49th annual convention of the American association of equine practitioners. p. 21–25.

[skag127-B11] Brunsting J et al 2019. Can the hoof be shod without limiting the heel movement? A comparative study between barefoot, shoeing with conventional shoes and a split-toe shoe. Vet J. 246:7–11. 10.1016/j.tvjl.2019.01.012 30902192

[skag127-B12] Cox DR. 1972. Regression models and life‐tables. J R Stat Soc Series B Stat Methodol. 34:187–202. 10.1111/j.2517-6161.1972.tb00899.x

[skag127-B13] Dabbene I et al 2018. Clinical findings and prognosis of interference injuries to the palmar aspect of the forelimbs in Standardbred racehorses: a study on 74 cases. Equine Vet J. 50:759–765. 10.1111/evj.12836 29603343

[skag127-B14] Dahl VE , Singer ER, Garcia TC, Hawkins DA, Stover SM. 2023. Hoof expansion, deformation, and surface strains vary with horseshoe nail positions. Animals (Basel). 13:1872. 10.3390/ani13111872 37889766 PMC10251877

[skag127-B15] Ducro BJ , Gorissen B, Eldik P, Back W. 2009. Influence of foot conformation on duration of competitive life in a Dutch Warmblood horse population. Equine Vet J. 41:144–148. 10.2746/042516408X363800 19418742

[skag127-B16] Hampson BA , de Laat MA, Mills PC, Pollitt CC. 2011. Evaluation of primary epidermal lamellar density in the forefeet of near-term fetal Australian feral and domesticated horses. Am J Vet Res. 72:871–876. 10.2460/ajvr.72.7.871 21728846

[skag127-B17] Higuchi H , Nakamura M, Kuwano A, Kasamatsu M, Nagahata H. 2005. Quantities and types of ceramides and their relationships to physical properties of the horn covering the claws of clinically normal cows and cows with subclinical laminitis. Can J Vet Res. 69:155–158. https://pubmed.ncbi.nlm.nih.gov/15971682/15971682 PMC1142185

[skag127-B18] Holmquist U. 2023. Mapping of prevalence, diagnostics and treatments of orthopedic injuries in Swedish trotting horses. Deptartment of Clinical Sciences. Swedish University of Agricultural Sciences. http://urn.kb.se/resolve? urn=urn: nbn: se: slu: epsilon-s-18666.

[skag127-B5851278] Knight PK, , Thomson PC. 2011. Age at first start and racing career of a cohort of Australian Standardbred horses. Aust Vet J. 89:325–330. 10.1111/j.1751-0813.2011.00816.x 21864303

[skag127-B19] Ley W , Pleasant RS, Dunnington E. 1998. Effects of season and diet on tensile strength and mineral content of the equine hoof wall. Equine Vet J. 30:46–50. 10.1111/j.2042-3306.1998.tb05121.x 9932093

[skag127-B20] Trot L ,. 2024. Des pratiques de déferrage raisonnées et raisonnables. https://www.letrot.com/actualites/des-pratiques-de-deferrage-raisonnees-et-raisonnables-20170 [2024-10-16].

[skag127-B21] Misztal I et al 2014. Manual for BLUPF90 family of programs. http://nce.ads.uga.edu/wiki/lib/exe/fetch.php?media=blupf90_all8.pdf [2024-01-08].

[skag127-B22] Moiroud C et al 2014.Incidence du déferrage sur l’usure du pied et le confort du cheval trotteur en course. AVEF (association des vétérinaires équins français) – conférence annuelle.

[skag127-B23] Ricard A , Blouin C. 2011. Genetic analysis of the longevity of French sport horses in jumping competition. J Anim Sci. 89:2988–2994. 10.2527/jas.2011-3931 21551348

[skag127-B24] Roepstorff L , Johnston C, Drevemo S. 2001. In vivo and in vitro heel expansion in relation to shoeing and frog pressure. Equine Vet J Suppl. 33:54–57. 10.1111/j.2042-3306.2001.tb05359.x 11721569

[skag127-B25] Roepstorff L , Johnston C, Drevemo S. 1999. The effect of shoeing on kinetics and kinematics during the stance phase. Equine Vet J. 31:279–285. 10.1111/j.2042-3306.1999.tb05235.x 10659269

[skag127-B26] Rouette J , Cockram MS, Sanchez J, MacMillan KM. 2021. Musculoskeletal injuries in Standardbred racehorses on Prince Edward Island. Can Vet J. 62:987–993. https://pubmed.ncbi.nlm.nih.gov/34475585/34475585 PMC8360321

[skag127-B27] Sargolzaei M , Iwaisaki H, Colleau JJ,. 2006. CFC: a tool for monitoring genetic diversity. 8th World Congress on genetics applied to livestock production August 13–18. p. 27–28.

[skag127-B28] Schoenfeld D. 1982. Partial residuals for the proportional hazards regression model. Biometrika. 69:239–241. 10.1093/biomet/69.1.239

[skag127-B29] Schwochow D et al 2025. RNA-seq analysis identifies key genes enhancing hoof strength to withstand barefoot racing in Standardbred trotters. BMC Genomics. 26:751. 10.1186/s12864-025-11814-4 40826322 PMC12363045

[skag127-B30] Sobczynska M. 2007. The effect of selected factors on length of racing career in Thoroughbred racehorses in Poland. Anim Sci Pap Rep. 25:131–141.

[skag127-B31] Solé M , Lindgren G, Bongcam‐Rudloff E, Jansson A. 2020. Benefits and risks of barefoot harness racing in Standardbred trotters. Anim Sci J. 91:e13380. 10.1111/asj.13380 32363779

[skag127-B32] Solé M et al 2017. Assessment of sportive longevity in Pura Raza Español dressage horses. Livest Sci. 203:69–75. 10.1016/j.livsci.2017.07.007

[skag127-B33] Spörndly-Nees E , Jansson A, Pökelmann M, Pickova J, Ringmark S. 2023. Chemical composition of horse hooves with functional qualities for competing barefoot. J Anim Sci. 101:skad346. 10.1093/jas/skad346 PMC10601914 37814393

[skag127-B34] Svensk Travsport. 2023a. Vanliga frågor och svar om travhälsans stallbackskontroller. https://www.travsport.se/siteassets/relaterade-dokument/hastvalfard/vanliga-fragor-och-svar-om-travarhalsans-stallbackskontroller.pdf? 407 [2025-06-07].

[skag127-B35] Svensk Travsport. 2023b. Travdatabasen, del 1 – hur ofta tävlar hästarna barfota runt om? https://www.travsport.se/arkiv/nyheter/2023/maj/travdatabasen-del-1-hur-ofta-tavlar-hastarna-barfota-runt-om/ [2023-12-21].

[skag127-B36] Svensk Travsport. 2024. Travarhälsan Checklista och kontrollvägledning– egenkontroll inför start. https://www.travsport.se/siteassets/relaterade-dokument/hastvalfard/checklista-och-vagledning.pdf? 407 [2025-06-07].

[skag127-B37] Svensk Travsport. 2025a. Tävlingsreglemente 2025. https://www.travsport.se/siteassets/regelverk/tavlingar/tavlingsreglemente.pdf? 537 [2025-06-01].

[skag127-B38] Svensk Travsport. 2025b. Svensk Travsports mall för påföljder. https://www.travsport.se/siteassets/regelverk/tavlingar/svensktravsports-pafoljdsmall.pdf? 111 [2025-12-14]

[skag127-B39] Svensk Travsport. 2025c. Avels- och registreringsreglemente 2025. https://www.travsport.se/siteassets/regelverk/avel-och-uppfodning/avels-och-registreringsreglemente.pdf?670 [2025-05-30].

[skag127-B40] Svensk Travsport. 2025d. Tävlingarna. https://www.travsport.se/svensk-travsport/travsporten-i-sverige/tavlingarna/ [2025-06-09].

[skag127-B41] The European Trotting Union. 2024. UET animal welfare regulations France. https://www.uet-trot.eu/en/regulations/? state=france#5844 [2025-02-06].

[skag127-B42] The European Trotting Union. 2025a. UET animal welfare regulations Germany. https://www.uet-trot.eu/en/regulations/? state=allemagne [2025-06-20].

[skag127-B43] The European Trotting Union 2025-06-20. 2025b. UET animal welfare regulations Italy. https://www.uet-trot.eu/en/regulations/? state=italie

[skag127-B44] The European Trotting Union 2025-06-20. 2025c. UET animal welfare regulations Finland. https://www.uet-trot.eu/en/regulations/? state=finlande

[skag127-B45] Therneau TM. 2024. A Package for Survival Analysis in R. https://CRAN.R-project.org/package=survival.

[skag127-B46] Therneau TM , Grambsch PM. 2000. The cox model. Modeling survival data: Extending the cox model. Statistics for Biology and Health, Springer. 10.1007/978-1-4757-3294-8_3

[skag127-B47] Velie B , Jäderkvist Fegraeus K, Ihler C, Lindgren G, Strand E. 2019. Competition lifespan survival analysis in the Norwegian‐Swedish Coldblooded Trotter racehorse. Equine Vet J. 51:206–211. 10.1111/evj.12989 29969157

[skag127-B48] Vigre H , Chriél M, Hesselholt M, Falk-Rønne J, Ersbøll AK. 2002. Risk factors for the hazard of lameness in Danish Standardbred trotters. Prev Vet Med. 56:105–117. 10.1016/S0167-5877(02)00158-7 12450683

[skag127-B49] Wilson A , Weller R. 2011. The biomechanics of the equine limb and its effect on lameness. In: Ross MW, Dyson SJ, editors. Diagnosis and management of lameness in the horse. 2nd ed. Elsevier/Saunders. p. 270–281. 10.1016/B978-1-4160-6069-7.00026-2

[skag127-B50] Yoshihara E et al 2010. Heel movement in horses: comparison between glued and nailed horse shoes at different speeds. Equine Vet J Suppl. 42:431–435. 10.1111/j.2042-3306.2010.00243.x 21059041

[skag127-B51] Zhang Y , Driedger C, Moussavi M, Tardieu EM. 2026. From fetus to fossil: a microscopic analysis of the chemistry of the hoof throughout the stages of life. McGill Bioengineering Hyperbook, Department of Bioengineering. https://bioengineering.hyperbok.mcgill.ca/from-fetus-to-fossil-a-microscopic-analysis-of-the-chemistry-of-the-hoof-throughout-the-stages-of-life/ [2026-02-24]

